# Evolutionary rescue from climate change: male indirect genetic effects on lay-dates and their consequences for population persistence

**DOI:** 10.1093/evlett/qrad022

**Published:** 2023-07-13

**Authors:** Myranda Murray, Jonathan Wright, Yimen G Araya-Ajoy

**Affiliations:** Centre for Biodiversity Dynamics (CBD), Department of Biology, Norwegian University of Science and Technology (NTNU), N-7491 Trondheim, Norway; Centre for Biodiversity Dynamics (CBD), Department of Biology, Norwegian University of Science and Technology (NTNU), N-7491 Trondheim, Norway; Centre for Biodiversity Dynamics (CBD), Department of Biology, Norwegian University of Science and Technology (NTNU), N-7491 Trondheim, Norway

**Keywords:** indirect genetic effect, cross-sex genetic correlation, adaptive landscape, quantitative genetics, sexual interactions, social evolution

## Abstract

Changes in avian breeding phenology are among the most apparent responses to climate change in free-ranging populations. A key question is whether populations will be able to keep up with the expected rates of environmental change. There is a large body of research on the mechanisms by which avian lay-dates track temperature change and the consequences of (mal)adaptation on population persistence. Often overlooked is the role of males, which can influence the lay-date of their mate through their effect on the prelaying environment. We explore how social plasticity causing male indirect genetic effects can help or hinder population persistence when female genes underpinning lay-date and male genes influencing female’s timing of reproduction both respond to climate-mediated selection. We extend quantitative genetic moving optimum models to predict the consequences of social plasticity on the maximum sustainable rate of temperature change, and evaluate our model using a combination of simulated data and empirical estimates from the literature. Our results suggest that predictions for population persistence may be biased if indirect genetic effects and cross-sex genetic correlations are not considered and that the extent of this bias depends on sex differences in how environmental change affects the optimal timing of reproduction. Our model highlights that more empirical work is needed to understand sex-specific effects of environmental change on phenology and the fitness consequences for population dynamics. While we discuss our results exclusively in the context of avian breeding phenology, the approach we take here can be generalized to many different contexts and types of social interaction.

## Introduction

Climate change alters the optimal timing of reproductive events of wild populations (Parmesan & Yohe 2003; Thackeray et al. 2010; Walther et al. 2002), and a classic example is the effect of spring warming on avian breeding phenology (Visser, 2008). In seasonal environments, reproductive timing (measured as the date the first egg is laid) acts as a gatekeeper for phenological synchrony between food abundance and the energetic demands of reproduction (Dunn et al., 2011). As climate change drives increasingly warmer springs, the emergence of resource species (e.g., arthropods) advances, and populations that do not adapt to new conditions can experience variation in population vital rates (Nolet et al., 2020; Reed et al., 2013; Regular et al., 2014; Visser & Gienapp, 2019) and population declines (Both et al., 2006; Ross et al., 2017). Meta-analyses suggest that the onset of breeding is under directional selection for earlier reproduction and has advanced over the years through a combination of microevolution and phenotypic plasticity (Charmantier & Gienapp, 2014; de Villemereuil et al., 2020; Dunn & Møller, 2014; Marrot et al., 2018; Radchuk et al., 2019). As global temperatures continue to rise, a key question intersecting conservation and evolutionary ecology is whether the timing of reproduction can adapt fast enough to “rescue” populations from extinction (Gienapp et al., 2013; Radchuk et al., 2019; Simmonds et al., 2020; Vedder et al., 2013).

Most studies on adaptation to climate change overlook the fact that, while lay-date is a trait expressed by females, the phenotype may be “shared” between the sexes because its expression necessarily involves an interaction with a mate. Because females inevitably interact with their breeding partner, their laying dates are likely affected by heritable traits expressed by their mate. Behavioral ecologists have extensive evidence on how social interactions with mates affect reproductive timing through courtship feeding, territory defense, territory quality, timing arrival at the breeding grounds, vocalizations, or any other behavior expressed after mate choice (Ball, 1993; Brockway, 1965; Brommer et al., 2015; Chmura et al., 2020; Griffith, 2019; Helm et al., 2006; Leboucher et al., 1998; Mitrus, 2006; Servedio et al., 2019; Setiawan et al., 2007; Shields et al., 1989; Waas et al., 2005; Whelan et al., 2016). Whenever the male phenotype has a genetic basis, female (social) plasticity creates an indirect genetic effect (IGE) where the male genotype affects the phenotypic expression of lay-date (Brommer & Rattiste, 2008; Evans et al., 2020; Germain et al., 2016; Liedvogel et al., 2012; Moirón et al., 2020; Teplitsky et al., 2010). Quantitative genetic IGE theory predicts that male trait evolution can affect the tempo and potentially the direction of evolution through social interactions (McGlothlin et al., 2010; Moore et al., 1997), leading to verbal arguments that IGEs provide an additional mechanism for climate change adaptation (Fisher et al., 2021; Pujol et al. 2018).

Lay-date may also be considered a “shared” trait because the sexes share part of their autosome, and genes may have pleiotropic effects for the factors influencing female lay-date and the factors influencing the timing of reproductive events in males (Bourret & Garant, 2015; Caprioli et al., 2012; Liedvogel et al., 2009; Zhang et al., 2017). Cross-sex genetic correlations reflect the common genetic basis of female and male phenotypic variation. Most cross-sex genetic correlations are positive (Poissant et al., 2010), which can hinder adaptation when the evolutionary interests of the sexes diverge and help adaptation when they align (Berger et al., 2014; Bonduriansky et al., 2008; Connallon & Hall 2016; Hangartner et al., 2022; Jones et al., 2004; Kane et al., 2022; Matthews et al., 2019; Walsh & Blows 2009). However, the evolutionary dynamics of lay-date can be more complicated when there is a cross-sex genetic correlation between female lay-date and male trait(s) mediating IGEs (Moore & Pizzari 2005; Pennell & Morrow 2013; Plesnar-Bielak & Łukasiewicz 2021).

Intuitively, predictions about whether birds can evolve fast enough to avoid demographic consequences of climate change may be biased if male effects on female lay-dates are overlooked. Ultimately, the question is not whether males influence lay-date, but rather which factors determine the magnitude of these effects on local adaptation and population persistence under increasingly warmer springs. To date, it remains unclear how IGEs can help or hinder population persistence, especially when the traits involved in the social interaction are genetically coupled. We use a trait-based approach to model responses to selection in both sexes, and we do so by extending current mathematical models of evolutionary rescue (moving optimum models: Bürger & Lynch, (1995); Chevin et al., (2010); Lynch & Lande, (1993) and deriving an equation for the maximum rate of temperature change the population can track. We evaluate the model using a combination of empirical estimates from wild bird populations and simulated data to explore how the evolutionary dynamics of male phenotypes in lay-date responses to climate change affect the predictions for population persistence. We discuss our findings in relation to key parameters and their estimation in the hopes of stimulating future empirical work.

## Methods

### Model description

**Figure 1.**
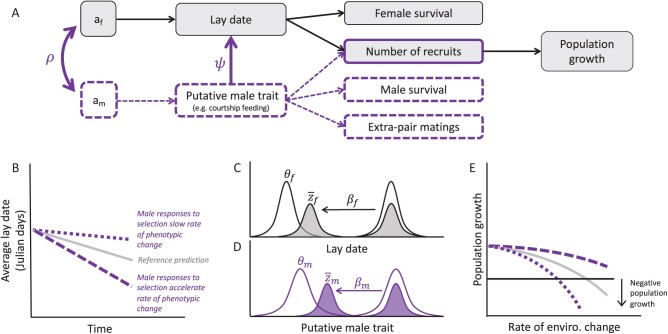
Schematic representation of the model. **(A)** The population dynamics consequences of the evolution of lay-date (*z*_*f*_) and the trait of male breeding partners (*z*_*m*_) can be broken down into a series of paths at different biological scales. Each trait has a additive genetic basis that affects its phenotype, which in turn affects vital rates and population growth. The classic sex-limited approach is shaded in gray. Male modifications of the genotype–phenotype relationship via the cross-sex genetic covariance (*G*_*fm*_), and female social plasticity in response to the male trait ($\psi$), are shown with dashed, purple lines. **(B)** Male effects on the genotype–phenotype map can bias the evolutionary trajectory upward or downward depending on the sign and magnitude of $\psi$ and the cross-sex genetic correlation $\rho$. **(C and D)** Each trait is characterized by a quadratic fitness function with phenotypic optimum $\theta$. The population is initially adapted to local conditions ($\theta=\bar{z}$) before the optimum phenotype advances with increasing spring temperatures, which generates directional selection ($\beta$) toward earlier reproduction. **(E)** Population growth is a function of the mismatch between the optimum lay-date and the average lay-date, and thus decreases with faster rates of environmental change. The “critical rate of environmental change” occurs when the average lay-date lags far enough behind its optimum that the population can no longer replace itself. Populations that can track their phenotypic optimum more closely can persist under higher rates of environmental change—see Equation 4, Table 1, and main text for more details.

The main components of moving optimum models can be represented schematically through a series of simplified paths that connect evolutionary processes to population growth (Figure 1), with Table 1 providing a full description of model parameters. In the following sections, we describe a basic quantitative genetic moving optimum model based on Chevin et al., (2010) that describes the evolutionary and demographic dynamics when lay-date is treated as a sex-limited female-only trait (in gray, Figure 1). Then we describe how this baseline model can be extended to also include male effects (in purple, Figure 1). For simplicity, we assume a density-independent panmictic population with constant additive genetic variance and no genetic drift, dispersal, or phenotypic plasticity, such that phenotypic change is caused solely by microevolution.

**Table 1.** Description of model parameters. $j=[f,m]$ is an index of female- and male-specific parameters, respectively.

Symbol	Description
*z*_*j*_	Phenotypic trait value
*G*_*j*_	(Direct) additive genetic variance
$\psi$	Social plasticity
*G*_*fm*_	Additive genetic covariance between female and male traits
$\rho$	Additive cross-sex genetic correlation between female and male traits $\rho=\frac{G_{fm}}{\sqrt{G_{f}G_{m}}}$
$\xi$	Cross-sex genetic regression ($\xi=\frac{\rho\sqrt {G_{f}G_{m}}}{G_{m}}$)
$\theta_{j}$	Optimum phenotype
$\eta$	Rate of environmental change
$\eta_{c}$	Critical rate of environmental change (beyond which the model predicts extinction)
*B*_*j*_	Rate of change of the optimum phenotype
$\gamma_{j}$	Stabilizing selection on trait *z*_*j*_
$\beta_{j}$	Directional selection on trait *z*_*j*_
*r*_*max*_	Intrinsic growth rate
*T*	Generation time

#### Moving optimum models and the critical rate of environmental change

The simplest scenario captures how a focal genotype affects the phenotypic value proportional to the (direct) additive genetic variance (*G*_*f*_) in a standard quantitative genetic genotype–phenotype map (Figure 1A). The phenotype can affect survival and the recruitment of new offspring to the population. The phenotype-vital rate map thus characterizes the natural selection gradients ($\beta_{f}$) acting on different fitness components. Population vital rates determine the mean fitness of the population ($\bar{W}$) or population growth rate.

Moving optimum models are characterized by two functions: a normally distributed phenotype (lay-date) with mean $\bar{z}_{f}$ and phenotypic variance $\sigma_{f}^{2}$, and a quadratic fitness landscape with peak $\theta_{f}$ that defines the phenotype that confers the highest fitness (herein the “optimum” phenotype). The widths of the fitness and phenotype functions are held constant, but their peaks shift (Figure 1C). The phenotype function shifts according to the expected generational change in the average lay-date predicted by the Lande equation $\Delta \bar{z}_{f} = \frac{1}{2}\beta_{f} G_{f}$, where the strength of selection is scaled by 0.5 because it is sex-specific (Lande, 1979). The fitness function shifts according to the change in the optimum phenotype, where $\Delta \theta = B\eta$. $\eta$ gives the rate of environmental change per unit time ($^{\circ}$ C/year), which is assumed constant such that temperature increases linearly over time. *B* is the slope of the optimum with respect to temperature, assuming a linear relationship, analogous to a linear reaction norm (Chevin et al., 2010). Between populations, the sensitivity of the optimum phenotype to temperature change can differ according to factors such as diet, prey distributions throughout the breeding season (Dunn et al., 2011), trade-offs between energetic demands of nestlings versus the energetic costs of earlier reproduction (Visser et al., 2012), or competitive interactions (Day & Kokko, 2015; Reed et al., 2013).

The population is initially adapted to local conditions ($\theta=\bar{z}$) and is under stabilizing selection around the optimum. The optimum lay-date advances as temperatures increase, which generates directional selection (Figure 1C). Responses to selection cause a proportional shift in the average lay-date and the population eventually reaches an equilibrium where the average lay-date tracks the shifting optimum at a constant rate.

At equilibrium, population growth is reduced by two genetic loads (Lande & Shannon, 1996). First is the standing load from stabilizing selection around the optimum lay-date, which increases with the phenotypic variance (and is thus assumed to be fixed under model assumptions). Second is the “lag load” (sensu Smith, 1976), which measures the reduction in population mean fitness as a function of the difference between the optimum and the average lay-dates (i.e., the degree of maladaptation; Figure 1E). As the lag load increases, the population eventually reaches a critical point defining the limit where it can no longer replace itself ($ln\bar{W}=0$). When this happens in the model, the rate of change in the average lay-date is demographically unsustainable and the population goes extinct.

A common output in moving optimum models is the critical rate of environmental change, which defines the maximum rate of environmental change the population can tolerate before it is expected to go extinct. Assuming discrete, nonoverlapping generations where population growth is a function of female adaptation, we can define a critical rate of environmental change for a reference scenario, where the evolutionary dynamics of the male trait do not affect female lay-dates:


\begin{equation*} \eta_{c} = \frac{1}{2} \sqrt{\frac{2r_{max}\gamma_{f}}{T}}\frac{G_{f}}{|B_{f}\,|}. \end{equation*} (1)

Processes that increase the critical rate of environmental change increase the population’s resilience to spring warming and thus population persistence, while processes that decrease it will increase the threat of extinction. The direct additive genetic variance (*G*_*f*_) and the strength of stabilizing selection ($\gamma_{f}$) have a positive effect on population persistence (Bürger & Lynch, 1995; Lynch & Lande, 1993). As a composite term, $\frac{r_{max}}{T}$ captures the pace-of-life of the population where “fast” species produce more offspring (higher *r*_*max*_) and have shorter generation times (lower *T*) than “slow” species that generally produce fewer offspring and live longer (see Gaillard et al., 2005; Wright et al., 2019). This implies that species with a faster pace-of-life have a higher critical rate of environmental change. Additionally, the critical rate of environmental change is affected by the pace at which the optimum lay-date shifts with spring temperature. The critical rate of environmental change ($\eta_{c}$) decreases with increasing sensitivity of the optimum lay-date to environmental change (*B*_*f*_) because higher rates of microevolution are needed to track a more rapidly moving optimum.

#### Model extensions

The model presented above relies on many simplifying assumptions, but we focus here on the implicit assumption that the genotype–phenotype map is characterized solely by the genes of the females that express the trait. In the following section, we describe how IGEs and cross-sex genetic correlations alter predictions about whether avian populations can evolve fast enough to avoid demographic consequences of climate change, using a series of extensions to Equation 1.

#### Male effects on the genotype–phenotype map

We extend the genotype–phenotype map to include a second phenotype expressed by male breeding partners (*z*_*m*_). The trait *z*_*m*_ can be timing of arrival at the breeding grounds, courtship feeding, or any other male phenotype that may affect lay-date (Figure 1A). Given the inherent time lag in the expression of each trait, the putative male trait affects lay-date, but not vice versa (i.e., males can affect a female’s lay-date, but lay-date cannot affect male prelaying behavior).

Sexual interactions give rise to IGEs when female lay-dates are affected by the phenotype expressed by their mate. This male trait is effectively a social environmental gradient whose effect on lay-date is captured by a linear reaction norm slope, $\psi$ (Dingemanse & Araya-Ajoy, 2015; Moore et al., 1997). Social plasticity in the female thus creates a pathway by which the male genotype affects (female) lay-date through $\psi G_{m}$. Predicted rates of change in the average lay-date will thus be affected by responses to selection in the male trait modulated by $\psi$. If the male trait is genetically independent from lay-date, this bias is $\psi\Delta\bar{z}_{m}=\psi\frac{1}{2}\beta_{m} G_{m}$ (Moore & Pizzari, 2005).

The genotype–phenotype relationship may be further complicated by genetic architecture of sex-specific traits involved in reproductive timing through cross-sex genetic covariance *G*_*fm*_. The cross-sex genetic covariance reflects the genetic association between sex-specific traits, where selection of one sex will have a corresponding response in the other. The effect of indirect selection on the average phenotype is expressed by $\frac{1}{2}\beta_{j} G_{fm}$, where $j=[f,m]$ is an index denoting sex.

Assuming no population structure, and constant values of $\psi$ and *G*_*fm*_, these two processes can be combined in a general equation that predicts generational change in the average lay-date:


\begin{equation*} \Delta\bar{z}_{f} = \frac{1}{2} (G_{f}\beta_{f} + G_{fm}\beta_{m} + \psi \underbrace{(G_{m}\beta_{m} + G_{fm}\beta_{f})}_{{\Delta \bar{z}_{m}}}) \end{equation*} (2)

(Moore et al., 1997; Moore & Pizzari, 2005), where all terms are as described above. The first composite term ($G_{f}\beta_{f}$) is the expected change in the average lay-date, proportional to the univariate Lande equation. The second composite term ($G_{fm}\beta_{m}$) describes the expected change in average lay-date due to indirect selection (i.e., the effect of direct selection on the male trait acting through any genetic covariance). The third and last term ($\psi (G_{m}\beta_{m} + G_{fm}\beta_{f})$) is the female’s plastic response to an evolving male trait ($\Delta \bar{z}_{m}$), which may itself evolve depending on any direct and/or indirect selection.

From Equation 2 (and IGE models in general), responses to selection in the male trait can cause changes in the average lay-date, even if there is no selection on lay-date itself. All else being equal, the contribution of males through $\psi\Delta\bar{z}_{m}$ will accelerate generational change in the average lay-date when aligned with the direction of direct selection on lay-date ($\beta_{f}$) or it will delay generational change when they are in the opposite direction (Figure 1b).

#### Consequences for population persistence

We now define separate fitness functions for female and male traits, with optimal values $\theta_{f}$ and $\theta_{m}$, respectively (Figure 1A and D). Environmental change is expected to advance the phenotypic optimum of each sex (De Lisle et al.), although sex-specific optima may advance at different rates owing to different energetic requirements, survival costs, and mating opportunities. This captures any potentially divergent evolutionary interests between the sexes, where the optimum lay-date and the optimum male trait change with spring warming proportional to $B_{f}\eta$ and $B_{m}\eta$, respectively, where $B_{f}B_{m} > 0$.

Both the female trait and the male trait track their shifting optima through microevolution and eventually reach an evolutionary equilibrium, which relies on the assumption that there is sufficient additive genetic variance in each trait to track the optimum. At equilibrium, the lag between the optimum phenotype and the population mean phenotype for each sex remains constant, with each trait shifting at rate $B_{j} \eta$ per unit time.

We assume female demographic dominance, where population dynamics are determined via female absolute fitness (which is a function of the difference between the optimum lay-date and the average lay-date). Therefore, any male effects on population growth occur indirectly by accelerating or slowing the pace of microevolutionary change in lay-date (Figure 1E). This assumption is a good approximation for the dynamics of populations with 50:50 sex ratios, greater female investment in offspring, and zero-sum mating (Crowley, 2000; Rankin & Kokko, 2007).

## Results

### Numerical evaluations

We extend the derivation of the equation for the critical rate of environmental change (Equation 1), starting with the effects of social plasticity causing IGEs on genetically independent traits, followed by the complications that can arise when interacting traits are genetically correlated. To illustrate how IGEs may affect the resilience of wild bird populations to temperature change, we evaluate the model using published parameter estimates and their error distributions (Table 2) under different genetic architectures and sensitivities to temperature change. More information and an interactive version of the simulation is available at http://myrandamurray.shinyapps.io/evorescuesimulations. Model derivations are provided in Supplementary Material.

**Table 2.** Summary of published parameter estimates ($\pm$*SE*) used in the simulations (Figure 3). *r*_*max*_ is the intrinsic growth rate of the population, *T* is age at first reproduction, *G*_*f*_ is the direct additive genetic variance, *G*_*i*_ the indirect additive genetic variance, and *G*_*fi*_ the direct-indirect genetic covariance. We back-calculate social plasticity under the simplifying assumption that male effects on lay-date are captured by a single male trait where $\psi=G_{fi}/G_i$.

Species	${\boldsymbol{r}}_{\boldsymbol{max}}$	*T*	$\boldsymbol{G}_{\boldsymbol{f}}$	$\boldsymbol{G}_{\boldsymbol{i}}$	$\boldsymbol{G}_{\boldsymbol{fi}}$	${\boldsymbol\psi}$
Common gull (*Larus canus*)	$0.12^{{\rm a}}$	$3.4^{{\rm b}}$	$4.52\,{\pm}\,1.20^{{\rm c}}$	$1.49\,{\pm}\,0.3^{{\rm c}}$	$-1.380\,{\pm}\,0.590^{{\rm c}}$	−0.93
Great tit (*Parus major*)	$0.49^{{\rm a}}$	$1.8\,{\pm}\,0.01^{{\rm d}}$	$5.96\,{\pm}\,0.94^{{\rm e}}$	$1.57\,{\pm}\,0.68^{{\rm e}}$	$1.011\,{\pm}\,0.583^{{\rm e}}$	0.64
Song sparrow (*Melospiza melodia*)	$0.77^{{\rm b,h}}$	$1.0^{{\rm f}}$	$12.30\,{\pm}\,0.370^{{\rm g}}$	$3.60\,{\pm}\,1.40^{{\rm g}}$	$6.588\,{\pm}\,0.00^{{\rm g}}$	1.8

a Niel & Lebreton (2005). b Rattiste & Lilleleht (1986). c Brommer & Rattiste (2008). d Vedder et al., (2013). e Evans et al., (2020). f Reid et al., (2019). g Germain et al., (2016). h Based on the rock sparrow (*Petronia petronia*).

We evaluate the model using an example where the IGE is caused by a trait reflecting the onset of male breeding behavior (e.g., when the male starts defending territory, provisioning, or singing). It follows from our scenario that any potential cross-sex genetic correlation between traits would likely be positive based on meta-analyses that show substantial positive cross-sex genetic correlation estimates between homologous traits (Poissant et al., 2010), with the general expectation that traits associated with sex-specific reproductive timing (e.g., controlling circadian rhythms and reproductive readiness) should have a shared genetic architecture and evolutionary history. Although we focus on sex-specific timing traits with optima that shift in the same direction and that are potentially positively correlated, our model can be generalized to any male trait that affects lay-date, optima that shift in opposing directions, and negative genetic correlations (see Supplementary Material).

#### Indirect genetic effects

Our first extension of the model accounts for male IGEs mediated by female social plasticity, assuming no cross-sex genetic correlations:


\begin{equation*} \eta_{c} = \frac{1}{2} \sqrt{\frac{2r_{max}\gamma_{f}}{T}}\frac{G_{f}}{|B_{f}-\psi B_{m}|}. \end{equation*} (3)

In extending Equation 1, we now include the term $\psi B_{m}$ that captures the contribution of IGEs to the critical rate of environmental change. This extension builds on prior models concerning partially adaptive phenotypic plasticity (Chevin et al., 2010). Phenotypic plasticity and social plasticity generating IGEs are analogous terms and are defined at the simplest level as linear reaction norm slopes across an environmental gradient. Phenotypic plasticity is generally considered in response to (a)biotic environmental gradients, whereas social plasticity is specific to variation in social environments created by the phenotype(s) of interacting conspecifics (i.e., the male phenotype in this case, Dingemanse & Araya-Ajoy, (2015)). A key distinction between models arises because the social environment can respond to selection. Under our assumption that the population reaches an evolutionary equilibrium, the effect of social plasticity on population persistence is modulated by the rate of change in the male optimum, *B*_*m*_. The term $\psi B_{m}$ thus describes how the rate of change in the environment affects the phenotypic mismatch between the optimum lay-date and the average lay-date through its effects on the optimal timing of reproduction for males.

**Figure 2.**
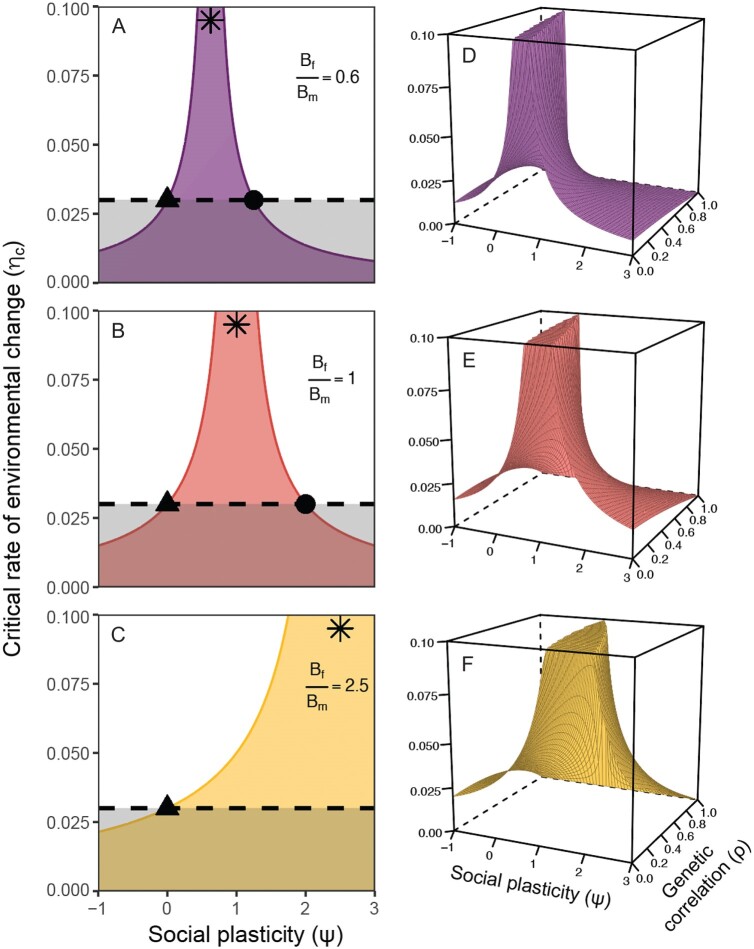
The effect of female social plasticity in response to the male trait ($\psi$) on the critical rate of environmental change ($\eta_{c}$) **(A–C)** when traits are genetically independent, that is, when the cross-sex correlation $\rho = 0$, and **(D–F)** when they are genetically coupled, that is, when $\rho\neq0$. Rows show different scenarios for the environmental sensitivity of the male optimum (*B*_*m*_) relative to that of the female optimum (*B*_*f*_): The male optimum is more sensitive to environmental change (top row); the male optimum and the optimum lay-date are equally sensitive to environmental change (middle row); and the male optimum is less sensitive to environmental change (bottom row). Asterisks indicate the degree of social plasticity that maximizes the critical rate of environmental change for each scenario. The horizontal dashed lines show the predicted critical rate of environmental change in the reference scenario when IGEs are not taken into account. The ranges of values of $\psi$ over which an IGE from breeding partners increases $\eta_{c}$ are marked with triangles (the lower limit) and circles (the upper limit). Parameter values: *r*_*max*_ = 0.5, *T* = 2, $\gamma = 0.005$, *G*_*f*_ = 6.0, *B*_*f*_ = −5.

Social plasticity shifts the average lay-date closer to the optimum phenotype as $|B_{f} - \psi B_{m}| \to 0$ and maximizes the critical rate of environmental change as $\psi$ approaches the ratio of environmental sensitivities of sex-specific optima, $\frac{B_{f}}{B_{m}}$. This reflects a scenario in our model where social plasticity allows the population track the shifting optimum almost entirely through IGEs (herein $\psi_{opt}$). Whenever the male optimum is less sensitive to environmental change than the optimum lay-date ($B_{f}/B_{m} > 1$), then the population persistence under constant environmental change is maximized at higher levels of social plasticity ($\psi_{opt} > 1$, Figure 2C), and vice versa when the male optimum is relatively more sensitive to environmental change ($B_{f}/B_{m} < 1$, $\psi_{opt}<1$, Figure 2A).

The contribution of IGEs to population persistence decreases nonlinearly as the average social plasticity in the population deviates from the optimal degree of social plasticity, and this can potentially hinder adaptive responses to climate change (points below the dashed line, Figure 2). Compared to a reference scenario that does not consider IGEs, social plasticity increases the critical rate of environmental change that can be demographically sustained whenever it is in the range $0 < \psi < 2B_{f}/B_{m}$, while social plasticity outside this range decreases the critical rate of environmental change. In our example, at the lower limit ($\psi \leq 0$), social plasticity causes populations to lag too far *behind* the optimum, due to a negative reaction norm slope that causes females to delay their lay-dates in response to changes in the male trait. At the upper limit of this range ($\psi \geq 2B_{f}/B_{m}$), social plasticity causes the average lay-date to trail far enough *ahead* of the optimum that the phenotypic mismatch between the optimum and the average lay-date is larger than it would have been in the absence of IGEs. In our example, negative social plasticity will always decrease the critical rate of change relative to the reference prediction, whereas positive social plasticity will only decrease the critical rate of environmental change when the contribution of IGEs ($\psi B_{m}$) to the change in lay-date per $^{\circ}$ C is more than double the sensitivity of optimum lay-date ($\psi B_{m} > 2B_{f}$).

**Figure 3.**
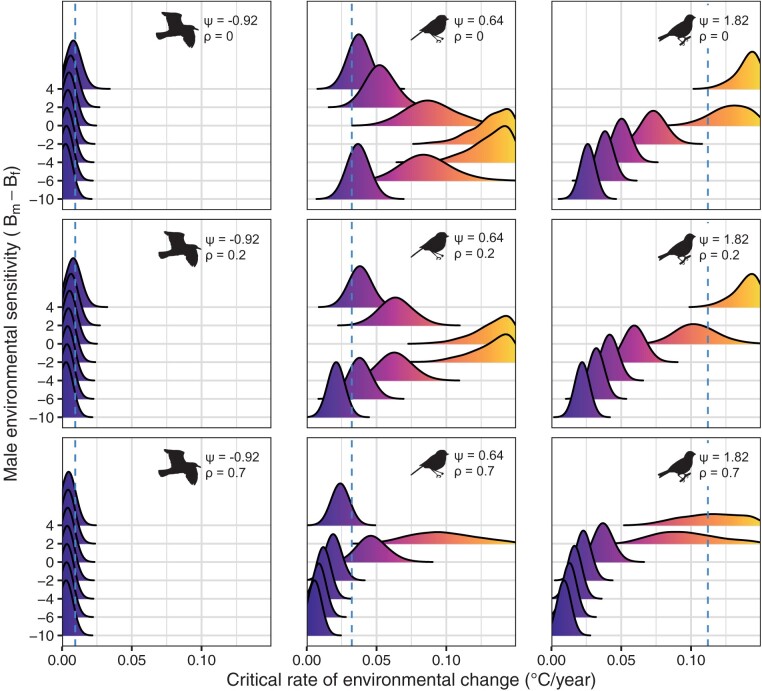
The effect of species-specific indirect genetic effects (IGEs) via social plasticity ($\psi$) on expected critical rates of environmental change ($\eta_{c}$) for common gulls (leftmost column), great tits (middle column) and song sparrows (rightmost column)—see Table 2 for parameter values. Results are shown for different scenarios of relative sensitivity of the male optimum (*B*_*m*_-*B*_*f*_, y-axis) and levels of pleiotropy in the cross-sex genetic correlation of male and female traits ($\rho$, the different rows). Environmental sensitivity of the male optimum (y-axis) is centered on the environmental sensitivity of the female optimum, where zero indicates that trait optima shift at the same rate, positive values indicate the male optimum lags behind the optimum lay-date, and negative values indicate that the male optimum advances more rapidly than the optimal lay-date. The color gradient corresponds to the critical rate of environmental change (x-axis). The vertical blue dashed line is the reference prediction when $\psi=\rho=0$. Densities are generated with 50,000 random samples using parameter estimates and their error distributions when available. For more information, see http://myrandamurray.shinyapps.io/evorescuesimulations.

Our simulations illustrate how the critical rate of environmental change can differ when IGEs are taken into account (Figure 3). The top row of Figure 3 (where $\rho = 0$) shows that for common gulls ($\psi = -0.93$), accounting for IGEs results in more pessimistic predictions for population persistence. When there is negative social plasticity, responses to selection for earlier breeding in the male trait have a delaying effect on the lay-date of their social breeding partner. For simulations based on parameters for great tits ($\psi = 0.64$), the critical rate of environmental change is higher than the reference prediction in nearly all of the parameter space we consider here, resulting in more optimistic predictions for population persistence. Males breeding earlier cause females to lay closer toward the optimum in this case, with the critical rate of environmental change being greatest when the male optimum is more sensitive to environmental change by approximately 2.5 days/$^{\circ}$ C (i.e., a one degree change in temperature shifts the male optimum 2.5 days earlier than the optimum lay-date). For the simulations based on song sparrows ($\psi = 1.8$), IGEs can increase or decrease predictions for population persistence depending on the sensitivity of the two sex-specific optima to temperature change. Owing to the greater degree of social plasticity in this population, the critical rate of environmental change is highest when the male optimum is less sensitive to environmental change by approximately 2 days/$^{\circ}$ C. At greater sensitivities in the male optimum, $\psi > 2B_{f}/B_{m}$, the critical rate of environmental change decreases, where IGEs cause females to lay too early.

#### Indirect genetic effects with genetically correlated traits

Sex-specific traits involved in the timing of reproduction are likely to exhibit strong cross-sex genetic correlations (Poissant et al., 2010). Hence, we further extend the model in this direction, building on prior work on intralocus sexual conflict (Connallon & Hall, 2016; Matthews et al., 2019). We can now express the full model predicting the critical rate of environmental change when lay-date is socially plastic and genetically correlated with the male trait causing the IGEs:


\begin{equation*} \eta_{c} = \frac{1}{2} \sqrt{\frac{2 \gamma_{f} r_{max}}{T}} \frac{G_{f}(1-\rho^{2})}{|B_{f}-(\psi + \xi) B_{m}|}, \end{equation*} (4)

where the cross-sex genetic correlation $\rho=G_{fm}/\sqrt{G_{f}G_{m}}$ (see Supplementart Material for derivation). For ease of comparison with the effects of social plasticity, we express the effect of the cross-sex genetic correlation on the denominator as a cross-sex regression coefficient (slope) $\xi = G_{fm}/G_{m} = \rho \sqrt{G_{f}}/\sqrt{G_{m}}$, which is analogous to the interaction coefficient $\psi$. Equation 4 captures how male trait evolution affect the mismatch between the average and optimum lay-dates. Reciprocal effects of selection in females on the male trait are subsumed into the term *B*_*m*_, which reflects the rate of change in the male trait per unit time at equilibrium. Given the assumption of female demographic dominance, the lag between the male trait and its optimum does not affect the limits of persistence.

From Equation 4, the cross-sex genetic correlation has a dual effect on the critical rate of environmental change. On one hand, it reduces sex-independent genetic variance proportional to $\frac{G_{fm}^{2}}{Gm}$. On the other hand, indirect selection from the correlated male trait can drag the average lay-date above or below its optimum phenotypic value depending on the cross-sex regression coefficient modulated by *B*_*m*_, much like the effects of social plasticity ($B_{m} \frac{G_{fm}}{G_{m}}$). The effects of IGEs from a genetically correlated trait on the critical rate of environmental change, therefore, depend on the contribution of indirect selection in the denominator and the reduction in sex-independent genetic variance in the numerator.

Figure 2D–F shows the effect of male trait evolution on the critical rate of environmental change, dependent on both the degree of social plasticity and indirect selection due to the cross-sex genetic correlation. The correlation between sex-specific traits introduces a second axis of variation in male effects on the critical rate of environmental change, such that the “optimal” degree of social plasticity can now be expressed as a function of the sensitivity ratio of sex-specific optima adjusted for the effect of indirect selection on the average lay-date, $\frac{B_{f}}{Bm}-\xi$. Under our scenario of a positive cross-sex genetic correlation, the genetic regression will be positive ($\xi>0$) and will therefore decrease the optimal degree of social plasticity (Figure 2D–F).

The range within which IGEs facilitate population persistence also changes when traits are genetically correlated (Figure 2D–F). The range within which IGEs increase the critical rate of environmental change, when compared with the reference prediction, can now be expressed as: $\frac{B_{f}\rho^{2}}{B_{m}}-\xi < \psi < \psi_{max}=2\frac{B_{f}(Bf-\rho^{2})}{B_{m}}-\xi$. Therefore, somewhat counterintuitively, if traits are strongly (and positively) genetically correlated, then populations with negative social plasticity may actually tolerate a higher rate of environmental change. Similarly, the effects of strong social plasticity that occur when IGEs drag the average lay-date too far ahead of the optimum are now also dependent on the cross-sex genetic correlation. Given the same sensitivity of the male optimum (*B*_*m*_), the average lay-date is more likely to be too early when both social plasticity and the genetic correlation are positive, compared to a scenario when there is no cross-sex genetic correlation (Equation 3).

Our simulations illustrate how the cross-sex genetic correlation can alter predictions of the critical rate of environmental change as a function of differences in the sensitivity of sex-specific optima (Figure 3). In the common gulls, accounting for cross-sex genetic correlations has little effect on the predicted outcome. Across all scenarios, the delaying effect of negative social plasticity on the average phenotype exceeds the advancing effect of indirect selection ($\psi< \frac{B_{f}\rho^{2}}{B_{m}}-\xi$), hindering population persistence. In the song sparrows and great tits, the critical rate of environmental change is highest with lower environmental sensitivity of the male optimum when compared with scenarios where the traits are genetically independent (e.g., for great tits, $B_{m}-B_{f} = -2.8$ days/$^{\circ}$ C when $\rho = 0$ vs. 1.3 days/$^{\circ}$ C when $\rho = 0.7$). Cross-sex genetic correlations also alter the range within which social plasticity facilitates adaptation. Continuing with the example of great tits, a cross-sex genetic correlation of 0.7 limits the adaptive range to only those scenarios in which $-0.6 < B_{m}-B_{f} < 3.1$. These changes are even more pronounced with increased cross-sex genetic correlations (i.e., $\rho=0.2$ vs. $\rho=0.7$, Figure 2D–F).

## Discussion

The ways in which bird species adjust their breeding phenology in response to climate change can affect population persistence, especially in light of widespread environmental change (Visser, 2008). Our models demonstrate that male responses to selection can be an important determinant of population adaptation when social interactions give rise to IGEs. This suggests that models that do not account for the shared nature of lay-date as a trait that is a product of selection on both sexes may provide biased results. Ultimately, whether IGEs help or hinder population adaptation depends on sexual differences in the environmental sensitivity of the optimum phenotype and cross-sex genetic correlations between traits (Figure 3).

While it is not surprising that IGEs in the opposite direction to the shifting optimal lay-date tend to hinder population adaptation in our model, a key insight emerging here is that IGEs in the direction of the shifting optimum can also hinder population-level adaptation. Because the contribution of IGEs to phenotypic change is modulated by the relative sensitivity of sex-specific optima, relatively strong selection on the male trait can lead to social plasticity causing females to lay earlier than is optimal (Servedio et al., 2019). This can, in some cases, result in much more pessimistic predictions for the critical rate of environmental change (Figure 3). It is thus of key importance to understand the evolutionary dynamics of the trait(s) causing the IGEs, otherwise their consequences for adaptive tracking of the optimum lay-date cannot be predicted. We therefore need a better understanding of sex-specific effects of climate change on the timing of reproduction (Williams et al., 2022). In addition to the commonly used variance-partitioning methods, this will require more trait-based models and empirical studies that investigate the male traits that affect female timing of reproduction and the fitness consequences this might have for males.

Our model also highlights the complications that occur when there are both IGEs and cross-sex genetic correlations (Figure 3). While IGEs and cross-sex genetic correlations arise from different mechanisms, they represent opposite ends of a continuum of male effects within and between loci (Abbott, 2011; Innocenti & Morrow, 2010; Pennell & Morrow, 2013). Populations are situated at some midpoint along this continuum when genetically correlated traits modulate social interactions and predictions concerning the eco-evolutionary consequences of one process depend on the strength and magnitude of the other. All else being equal, positive cross-sex genetic correlations between lay-date and the putative male trait are predicted by our model to increase the potential for evolutionary rescue at lower degrees of social plasticity and to create the potential for population persistence even with negative social plasticity that alone would decrease mean fitness (Figure). Our results align with integrative models of sexual conflict where simultaneous effects of (antagonistic) sexual interactions and pleiotropy between traits involved in sexual interactions can reciprocally exaggerate or mask the effects of each other (Berger et al., 2016; Pennell et al., 2016; Svensson, 2019).

Quantitative genetic moving optimum models are a useful tool to study the relative contribution of different processes underpinning adaptive responses to environmental change, but like any model they rely on simplifying assumptions (Carlson et al., 2014). Lay-date is a complex trait that depends on multiple (a)biotic cues (Dore et al., 2018; Dunn, 2019) and is likely correlated with other traits (e.g., clutch size, Evans et al., 2020; Sheldon et al., 2003). Competition for resources can also cause density-dependent changes in population size, and in such cases, any predicted outcome for population persistence might also depend on how density regulation is acting on the population (for discussion and references, see Osmond & de Mazancourt, 2013). Furthermore, selection itself may be dependent on the social environment through density-dependent, frequency-dependent, and/or other social selection (Formica et al., 2011; Gamelon et al., 2018; Svensson & Connallon, 2019). We have omitted these complications to focus on the relative contribution of social male effects. Including these additional processes could further alter any predictions for evolutionary rescue (reviewed in Bell 2017; Kopp and Matuszewski, 2014), although they are unlikely to affect our general conclusions regarding the relative contribution of male IGEs.

Responses to selection are just one way in which sexual interactions with a mate may affect any adaptation to climate change. Another way in which IGEs may influence responses to climate change is through the evolution of $\psi$ itself (Bailey & Zuk, 2012; Chenoweth et al., 2010; Rebar et al., 2020; Signor et al., 2017). A critical simplifying assumption of our model is the fixed values of certain key evolutionary parameters like $\psi$, but in natural systems, we might expect additive genetic (co)variances and selection gradients on social plasticity (Araya-Ajoy et al., 2020; Steppan et al., 2002). If $\psi$ can evolve, the contribution of social partners to population persistence would depend not only on responses to selection in the social trait, but also on the pace and direction of the concurrent evolution of $\psi$, which may not be in the same direction (Kazancıoğlu et al., 2012; Kuijper & Hoyle, 2015).

We also assume that mating is random, despite the fact that many natural systems may experience phenotypic assortment (e.g., based on timing of arrival at the breeding grounds, Moirón et al., 2020). In annually flowering plants, temporal assortative mating is predicted to contribute to evolutionary rescue by increasing additive genetic variance and facilitating more rapid tracking of the optimum (e.g., Godineau et al., 2021). Nonrandom assortment may also affect responses to selection because it creates a covariance between phenotypes (and genotypes) whereby any effects of the male phenotype on female fitness will be correlated with lay-date and can contribute to the net selection affecting evolutionary dynamics (Araya-Ajoy et al., 2020; Brodie et al., 2022; McGlothlin et al., 2010). Our model hopefully provides a useful framework that could be extended to include these kinds of additional social evolutionary processes that may influence rescue.

Throughout we have focused on the specific example of how birds adjust their timing of reproduction in response to climate change. However, our model can be generalized to a range of different scenarios of environmental change (e.g., desertification, pollution, habitat degradation) and types of nonreciprocal dyadic social interaction (e.g., between parents and offspring, mates, and competitors), and various different types of traits and species. IGEs are expected to be ubiquitous given the myriad traits that interact through competition, cooperation, communication and reproduction, and thus IGEs should be a common mechanism underpinning adaptive responses to environmental change (Araya-Ajoy et al., 2020; Fisher & McAdam, 2019; Garant, 2020; McAdam et al., 2014; Ramakers et al., 2018). Therefore, expanding the framework we present here to other contexts should greatly help improve our understanding of the eco-evolutionary implications of social interactions. We hope this model will stimulate the type of empirical work needed to estimate the key parameters we have outlined and also generate new questions that will bridge the divide between social evolution and conservation.

## Conclusions

Evolutionary conservation is concerned with the adaptive potential of species and populations, motivated by observations of phenotypic change on time span of generations. Understanding the processes that help or hinder adaptation, along with their demographic consequences, can help predict impacts of climate change on biodiversity, set emissions targets, and identify populations at risk of extinction. Our model contributes to growing awareness of the role of social environments in this respect, showing that avian lay-dates as phenotypes expressed by females may be influenced by both ecological and evolutionary effects of their male breeding partner, which can bias predictions for population persistence under warmer springs. Our model draws a direct link from social evolution theory on IGEs to evolutionary conservation and suggests that better integration of these two fields can be achieved by drawing attention to the explicit links between the evolution of conspecific interactions within populations and their effects on population dynamics, with the identification of key parameters acting as a guide for future empirical efforts.

## Supplementary Material

qrad022_suppl_Supplementary_Material

## Data Availability

There are no data associated with this article. Model derivations and simulation code are provided in Supplementary Material.
