# Supplementary material for: Evolutionary rescue from climate change: male indirect genetic effects on lay-dates and their consequences for population persistence
Source: Evol Lett. 2023 Jul 13;8(1):137–48. doi: 10.1093/evlett/qrad022 (PMC10939382; doi:10.1093/evlett/qrad022)
Supplement: qrad022_suppl_Supplementary_Material [file qrad022_suppl_supplementary_material.pdf]

Supporting Information for *Evolutionary rescue from climate change: male indirect genetic effects on lay-dates and their consequences for population persistence*

Myranda Murray, Jonathan Wright, Yimen Araya-Ajoy

Contents

1 Model derivations **1**
1.1 Temperature effects on fitness . . . 1
1.2 Responses to selection . . . 2
1.3 Population persistence . . . 2

2 Negative cross-sex genetic correlations **4**

1 Model derivations

Here we derive the critical rate of environmental change for lay-date when responses to selection are partially determined by evolution of a single trait expressed by male breeding partners. Let $j = [f, m]$ be an index indicating sex and z_j be the sex-specific phenotypes. We follow standard quantitative genetic assumptions (Falconer & Mackay, 1996) where both traits are normally distributed with mean $\bar{z}_j$ and variance σ_j^2, which we assume to be constant.

1.1 Temperature effects on fitness

Assuming there is no cost to social plasticity, the expected fitness of individuals expressing phenotype z_j can be expressed as:

$$W(z_j) = W_{max} exp - \frac{(z_j - \theta_j)^2}{2\omega_j^2} \quad , \tag{S1}$$

where W_{max} is the maximum fitness for an individual of the j'th sex expressing the optimum phenotype. W_{max} decreases as the square distance between the expressed phenotype and the sex-specific optimum phenotype increases (the phenotypic lag, $z_j - \theta_j$), and the width of the fitness function (ω_j).

The mean fitness of each sex is the partial integral of the product of the fitness function and the phenotypic function:

$$\bar{W}_j = \int W(z_j)p(z_j)dz = W_{max} \frac{\omega_j^2}{\sqrt{\omega_j^2 + \sigma_j^2}} exp - \frac{(\bar{z}_j - \theta_j)^2}{2\omega_j^2 + 2\sigma_j^2} \quad , \tag{S2}$$

(Chevin et al., 2010) where W_{max} is the maximum absolute fitness when every individual of sex j expresses the optimal phenotype, which is reduced by phenotypic variation (σ_j^2) around the optimum (the standing load Lande & Shannon, 1996) and the phenotypic distance to the optimum (the lag load Smith, 1976).

Selection on each sex captures how changes in mean phenotype result in changes in log mean fitness, which can be expressed in terms of the phenotypic lag:

$$\beta_j = \frac{\partial ln \bar{W}_j}{\partial \bar{z}_j} = \gamma(\bar{z}_j - \theta_j) \quad , \tag{S3}$$

(Lande, 1976) where $\gamma = 1/\omega_j^2 + \sigma_j^2$ is the strength of stabilizing selection around the optimum phenotype. From Equation S3, the strength of directional selection increases the farther the average phenotype deviates from the optimum phenotype. Assuming that temperature changes linearly at rate η, the optimum phenotype shifts at rate $B_j\eta$, where B_j is the environmental sensitivity of the optimum phenotype (following the notation of Chevin et al., 2010), which generates directional selection in response to environmental change.

1.2 Responses to selection

Responses to selection in the female trait are partially determined by an IGE from male breeding partners. The expected change in the average lay-date per unit time is:

$$\frac{\Delta \bar{z}_f}{T} = \frac{G_f\beta_f + G_{fm}\beta_m + \psi(G_m\beta_m + G_{fm}\beta_f)}{2T} \quad , \tag{S4}$$

and the expected change in the average male trait is:

$$\frac{\Delta \bar{z}_m}{T} = \frac{G_m\beta_m + G_{fm}\beta_f}{2T} \quad , \tag{S5}$$

where T is a measure of generation time in years and the remainder of the equations are as described in the main text.

Both sexes are initially adapted to local conditions ($z_j = \theta_j$) when the environment begins to change, causing the optimum phenotype for both sexes to advance. Over time, the population reaches an equilibrium where the phenotype z_j tracks its optimum at constant rate $B_j\eta T$ per generation. Substituting $\Delta\bar{z}_j/T = B_j\eta$) into Equation S4 and Equation S5, where $G_j \neq 0$, the phenotypic lag at equilibrium can be expressed for each sex as:

$$(\bar{z}_f - \theta_f)_{eq} = \frac{\eta T}{G_f\gamma_f(1-\rho^2)}(B_f - \psi B_m - \frac{G_{fm}}{G_m}B_m) \quad , \tag{S6}$$

and

$$(\bar{z}_m - \theta_m)_{eq} = \frac{\eta T}{G_m\gamma_m(1-\rho^2)}(B_m - \frac{G_{fm}}{G_f}B_f) \quad . \tag{S7}$$

The equilibrium lag is a measure of maladaptation, it quantifies how far the population mean trait deviates from its optimum. Equation S6 describes how the equilibrium lag of females depends, in part, on responses to selection in the male trait through the strength of IGEs and the cross-sex genetic correlation, while Equation S7 describes how the equilibrium lag of males is partially determined by indirect selection from females.

1.3 Population persistence

We model a density-independent population where population size from one time step to the next is given by $N_{t+1} = \bar{W}N_t$. Mean fitness greater than one indicates that the population size increases, mean fitness less than one indicates that the population size decreases and mean fitness of one indicates that the population is just replacing itself.

We assume that population growth is a function of the lag between the optimum and the average lay-date, and thus determined by the mean fitness of females who express the trait (female demographic dominance; Crowley, 2000; Rankin & Kokko, 2007). The growth rate of the population at a given time point can then be expressed a function of equilibrium female lag:

$$r = \frac{\ln \bar{W}_f}{T} = r_{max} - \frac{\gamma_f}{2T}(\bar{z}_f - \theta_f)^2_{eq} \tag{S8}$$

(Chevin et al., 2010). r_{max} is the maximum intrinsic growth rate when every individual expresses the optimum phenotype. Given the assumption of female demographic dominance, male (mal)adaptation does not affect the population growth rate (Equation S8). Rather, male effects on the population growth rate are realized indirectly through their effect on the female equilibrium lag (Equation S6), which introduces the potential for a female-biased fitness costs of selection.

To determine the limits of population persistence, we are interested in the maximum degree of maladaptation the population can tolerate while still being able to replace itself. Substituting Equation S6 into Equation S8 and setting $r = 0$ we then solve for the critical rate of environmental change beyond which the model predicts extinction:

$$\eta_c = \sqrt{\frac{2r_{max}\gamma_f}{T}} \left(\frac{G_f(1-\rho^2)}{|B_f - B_m(\psi + \frac{G_{fm}}{G_m})|} \right) \quad , \tag{S9}$$

where

$$r_{max} = \frac{\ln W_{max}}{T} - \frac{\ln(1+\sigma_f^2/\omega_f^2)}{2T} \quad . \tag{S10}$$

is reduced by phentoypic variation around the optimum, which we assume to be constant. See main text for more details.

2 Negative cross-sex genetic correlations

In the main text, we evaluate the model (Equation 4) under scenarios of positive cross-sex genetic correlations. Here, we explore the consequences of negative cross-sex genetic correlations, building upon prior work in intralocus sexual conflict in evolutionary rescue (Connallon & Hall, 2016; Hangartner et al., 2022; Kane et al., 2022; Matthews et al., 2019). We continue with our assumption that environmental change aligns the direction of selection between the sexes (De Lisle et al., 2018).

Recall that the genetic correlation has a dual effect on critical rate of environmental change: (1) it reduces the sex-independent additive genetic variance (proportional to $\frac{G_{fm}^2}{Gm}$ in the numerator), and (2) indirect selection shifts the average lay date closer to or farther from its optimum (according to ξB_m in the denominator). Without indirect genetic effects (IGEs), a negative cross-sex genetic correlation decreases the critical rate of environmental change compared to the reference scenario (where $\rho = \psi = 0$) by simultaneously reducing sex-independent genetic variance in the numerator and shifting the average lay date in the direction opposite to its shifting optimum. The critical rate of environmental change will always be lower than the reference prediction and decreases with the magnitude of the negative genetic correlation (panel B, Figure S1).

In scenarios with IGEs and negative cross-sex genetic correlations, the additive genetic variance is reduced in the numerator, but the effect of indirect selection depends on the sum of the genetic regression coefficient and the female's social plasticity to the evolving male trait. When there is a negative cross-sex genetic correlation and social responsiveness is also negative, the predicted critical rate of environmental change is even more pessimistic (panel A, Figure S1). However, when social responsiveness is positive, the demographic consequences caused by the delaying effects of the negative cross-sex genetic correlation are minimized or even masked when females advance their laying dates in response to their partner's phenotype (panels C and D, Figure S1).

The same general solutions described in the main text hold for scenarios with negative cross-sex genetic correlations. The critical rate of environmental change is greatest as ψ approaches $\frac{B_f}{Bm} - \xi$ (stars, Figure S2). Thus, the stronger the negative genetic correlation, the higher the degree of social plasticity needed to maintain a high critical rate of environmental change.

Social plasticity increases the critical rate of environmental change from the reference prediction whenever $\frac{B_f \rho^2}{B_m} - \xi < \psi < \psi_{max} = 2\frac{B_f(Bf-\rho^2)}{B_m} - \xi$. This implies that populations with a stronger negative genetic correlation will have a higher chance of persistence at higher degrees of social plasticity (Figure S2). Thus, even though there may be a negative cross-sex genetic correlations that, alone, would cause a lower critical rate of environmental change, the presence of IGEs can reduce or even counteract these this effect depending on the sign and magnitude of ψ and the relative sensitivities of sex-specific optima.

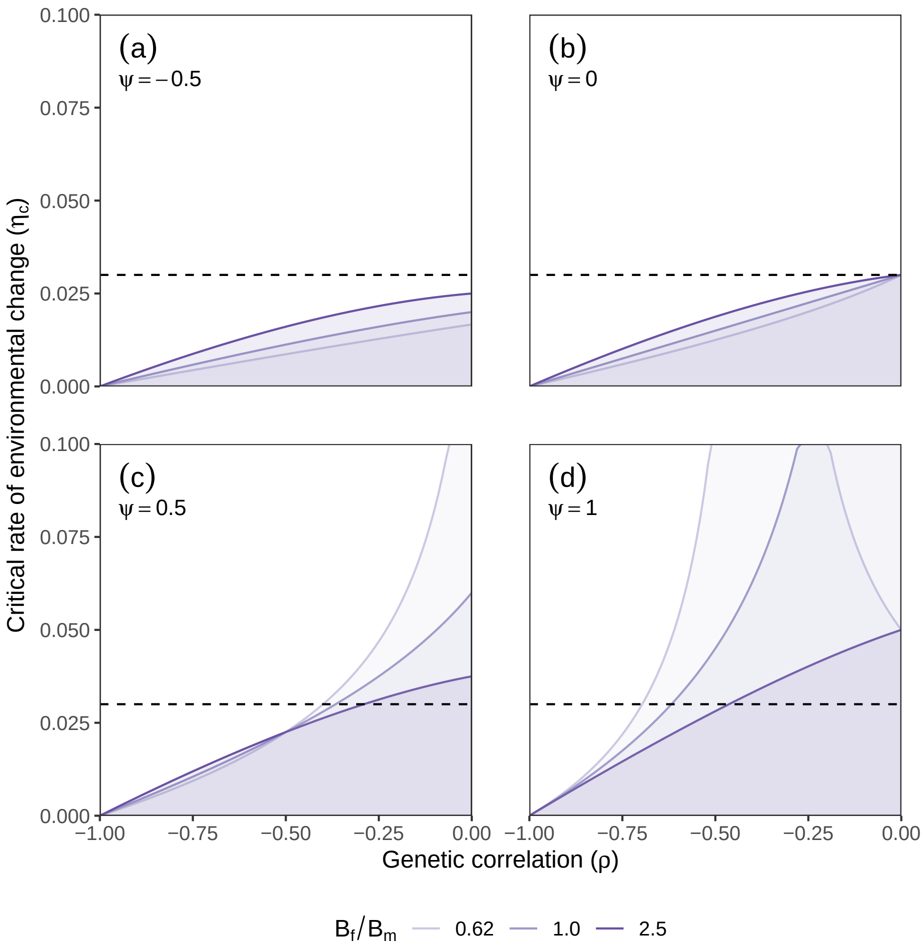

Figure 1: The effect of negative cross-sex genetic correlations on the critical rate of environmental change (η_c). The panels show different scenarios of social plasticity and colours represent different ratios of environmental sensitivity of sex-specific optima (B_f/B_m). The horizontal dashed line shows the reference prediction for the critical rate of environmental change when males are not taken into account ($\psi = \rho = 0$). Parameter values: $r_{max} = 0.5$, $T = 2$, $\gamma = 0.005$, $G_f = G_m = 6.0$, $B_f = -5$.

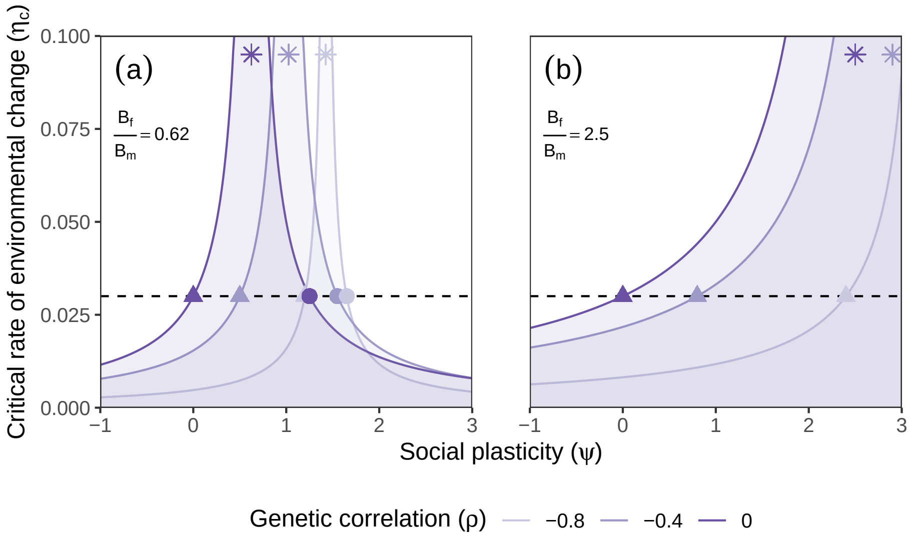

Figure 2: The effect of indirect genetic effects on the critical rate of environmental change (η_c) with different cross-sex genetic correlations (shown with different colours). Panels show different scenarios different ratios of environmental sensitivity of sex-specific optima (B_f/B_m). The horizontal dashed line shows the reference prediction for the critical rate of environmental change when males are not taken into account ($\psi = \rho = 0$). Stars show the optimal degree of social plasticity that leads to the highest critical rate of environmental change for a given scenario. The range of social plasticity that increases the critical rate of environmental change from the reference is shown by triangles (lower limit) and circles (upper limit). Parameter values: $r_{max} = 0.5$, $T = 2$, $\gamma = 0.005$, $G_f = G_m = 6.0$, $B_f = -5$.

References

Chevin, L.-M., Lande, R., & Mace, G. M. (2010). Adaptation, plasticity, and extinction in a changing environment: Towards a predictive theory. *PLoS Biol.*, *8*(4), e1000357.

Connallon, T., & Hall, M. D. (2016). Genetic correlations and sex-specific adaptation in changing environments. *Evolution*, *70*(10), 2186–2198.

Crowley, P. H. (2000). Sexual dimorphism with female demographic dominance: Age, size, and sex ratio at maturation. *Ecology*, *81*(9), 592–2605.

De Lisle, S. P., Goedert, D., Reedy, A. M., & Svensson, E. I. (2018). Climatic factors and species range position predict sexually antagonistic selection across taxa. *Philos. Trans. R. Soc. B Biol. Sci.*, *373*(1757), 20170415.

Falconer, D., & Mackay, T. F. C. (1996). *Introduction to quantitative genetics* (4th ed.). Longman.

Hangartner, S., Sgrò, C. M., Connallon, T., & Booksmythe, I. (2022). Sexual dimorphism in phenotypic plasticity and persistence under environmental change: An extension of theory and meta-analysis of current data. *Ecol. Lett.*, ele.14005.

Kane, A., Ayllón, D., O'Sullivan, R. J., McGinnity, P., & Reed, T. E. (2022). Escalating the conflict? Intersex genetic correlations influence adaptation to environmental change in facultatively migratory populations. *Evol. Appl.*, *15*(5), 773–789.

Lande, R. (1976). Natural selection and random genetic drift in phenotypic evolution. *Evolution*, *30*(2), 314–334.

Lande, R., & Shannon, S. (1996). The role of genetic variation in adaptation and population persistence in a changing environment. *Evolution*, *50*(1), 434–437.

Matthews, G., Hangartner, S., Chapple, D. G., & Connallon, T. (2019). Quantifying maladaptation during the evolution of sexual dimorphism. *Proc. R. Soc. B Biol. Sci.*, *286*(1908), 20191372.

Rankin, D. J., & Kokko, H. (2007). Do males matter? The role of males in population dynamics. *Oikos*, *116*(2), 335–348.

Smith, J. M. (1976). What determines the rate of evolution? *Am. Nat.*, *110*(973), 331–338.
